# Effect of daridorexant on wakefulness throughout the night: Post-hoc analysis of a randomised, double-blind, active reference (zolpidem) study in patients with insomnia disorder

**DOI:** 10.1192/j.eurpsy.2025.511

**Published:** 2025-08-26

**Authors:** B. Steiniger-Brach, O. Briasoulis, A. Olivieri, S. Pain, L. Palagini, D. Kunz, P.-A. Geoffroy

**Affiliations:** 1 Idorsia Pharmaceuticals Ltd., Allschwil, Switzerland; 2 Department of Neuroscience, Section of Psychiatry, University of Pisa, Pisa, Italy; 3 Clinic for Sleep & Chronomedicine, St. Hedwig-Krankenhaus, Große Hamburger Straße 5-11, 10115, Berlin, Germany; 4 GHU Paris - Psychiatry & Neurosciences; 5 Université de Paris, NeuroDiderot, Inserm; 6 Département de Psychiatrie et d’Addictologie, AP- HP, GHU Paris Nord, DMU Neurosciences, Hôpital Bichat - Claude Bernard, Paris, France

## Abstract

**Introduction:**

Daridorexant, a dual orexin receptor antagonist [DORA] which works by selectively reducing the orexin-induced wake signalling, has been shown to induce a dose-dependent reduction in wake time after sleep onset [WASO] in patients with insomnia disorder (Dauvilliers et al. Ann Neurol 2020; 87 347–356).

**Objectives:**

This exploratory analysis examined the efficacy of daridorexant in reducing the duration of awakenings in each quarter of the night, when compared to placebo and to the GABA-receptor agonist zolpidem, which induces sleep through widespread CNS sedation.

**Methods:**

This was a multi-centre, double-blind trial (NCT02839200), including adult (18–64y) patients with insomnia randomized (1:1:1:1:1:1) to placebo, daridorexant (5, 10, 25, or 50mg), or zolpidem (10mg) for 30 days. Polysomnography [PSG]-determined WASO was evaluated using descriptive statistics by quarter of the night (Q1–Q4) i.e. every 2 hours over 8 hours at Days 1 & 2, 15 & 16, and 28 & 29. Baseline was defined as the mean of the two PSG nights during the run-in period and Days 1&2 as the mean of the first two PSG treatment nights; Days 15&16 and 28&29 were defined similarly.

**Results:**

Dose-dependent decreases in mean change from baseline in Q1–Q4 WASO were observed with daridorexant (5–50mg) at Days 1 & 2 (** Figure 1**). Whereas the approved doses of daridorexant (25mg and 50mg) provided similar response to zolpidem 10mg in the first half of the night, mean reductions from baseline in WASO were numerically greater with daridorexant 50mg versus zolpidem 10mg during the second half of the night – with the difference most pronounced in the fourth quarter (mean WASO change from baseline Q3: –13.49 min versus –9.73 min; Q4: –17.51 min versus –7.81 min). Similar effects were seen at Days 15 & 16, and Days 28 & 29.

**Image 1:**

**Figure:**
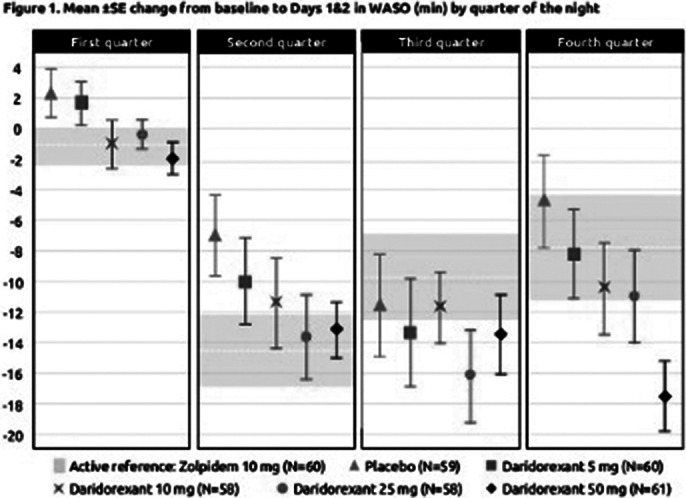

**Conclusions:**

In patients with insomnia disorder, daridorexant reduces the duration of awakenings throughout the entire night, including the last two quarters.

**Disclosure of Interest:**

B. Steiniger-Brach Employee of: Idorsia Pharmaceuticals, O. Briasoulis Employee of: Idorsia Pharmaceuticals, A. Olivieri Employee of: Idorsia Pharmaceuticals, S. Pain Employee of: Idorsia Pharmaceuticals, L. Palagini Consultant of: Bruno, Fidia, Idorsia Pharmaceuticals, Pfizer, Sanofi, Pharmanutra, Neopharmed Gentili, D. Kunz Consultant of: Austrian Association of Skiing (ÖSV), Idorsia Pharmaceuticals, Speakers bureau of: AbbVie, Idorsia Pharmaceuticals, German Ministry for Economy (BMWi), Austrian Association of Skiing (ÖSV), P.-A. Geoffroy Consultant of: Apneal, Arrow, Biocodex, Dayvia, Di&Care, Idorsia Pharmaceuticals, Janssen-Cilag, Jazz pharmaceuticals, Myndblue, Mysommeil, Posos, ResilEyes, Withings, Speakers bureau of: Biocodex, Bioprojet, Ibsa, Idorsia Pharmaceuticals, Janssen-Cilag, Isis Medical, Jazz pharmaceuticals, Lundbeck, MySommeil, Withings.

